# Exploration of the application of Grey-Markov models in the causality analysis of traffic accidents in roundabouts

**DOI:** 10.1371/journal.pone.0287045

**Published:** 2023-09-28

**Authors:** Peijing Li, Jian Li

**Affiliations:** 1 Department of Electrical Engineering and Computer Science, University of Michigan, Ann Arbor, Michigan, United States of America; 2 College of Fashion and Design, Donghua University, Shanghai, China; Tsinghua University, CHINA

## Abstract

We propose a multivariate Grey-Markov model to quantify traffic accident risk from different causality factors in roundabouts that is uniquely suited for the scarce and stochastic traffic crash data from roundabouts. A data sample of traffic crashes occurring in roundabouts in the U.S. State of Michigan from 2016 to 2021 was collected to investigate the capabilities of this modeling methodology. The multivariate grey model (MGM(1,4)) was constructed using grey relational analysis to determine the best dimensions for model optimization. Then, the Markov chain is introduced to address the unfitness of stochastic, fluctuating data in the MGM(1,4) model. Finally, our proposed hybrid MGM(1,4)-Markov model is compared with other models and validated. This study highlights the superior predictive performance of our MGM(1,4)-Markov model in fore-casting roundabout traffic accidents under data-limited conditions, achieving a 3.02% accuracy rate, in contrast to the traditional GM(1,1) model at 8.30% and the MGM(1,4) model at 4.47%. Moreover, incorporating human, vehicle, and environmental risk factors into a multivariate crash system yields more accurate predictions than merely aggregating crash counts.

## Introduction

Modern roundabouts require traffic from all sides to yield circulating traffic, with sufficient horizontal curvature to slow entry and circulation speeds, and they are widely touted as an effective safety intervention to replace traditional intersections to lower accident rates and severities. This is in part due to their reduction in the number and types of conflicts between vehicles and/or pedestrians moving in different directions through the intersection [1, 2]. In the meantime, forecasting roundabout traffic accidents and identifying factors determining the severity of injuries to road users is an essential component of accident prevention decision-making and avoiding casualties or damage. However, the safety performance at roundabouts is affected by many uncertainty factors, such as the interaction of collision factors—humans, vehicles, and road environment- which negatively exhibits uncertain variability and ultimately complicates the performance prediction of roundabout traffic [3, 4]. Hence efficient methods are needed to capture this uncertainty, predict roundabout traffic crashes accurately beyond empirical observations, and analyze their causality quantitatively, ideally with the help of emerging data collection and processing techniques, to inform future transportation policy and planning.

The most significant issue with modeling and predicting traffic crashes in roundabouts is the scarcity and stochasticity in the data for historical roundabout traffic accidents. This characteristic in such datasets, combined with the aforementioned fact that a variety of human, vehicle, and environmental factors all affect the safety performance of roundabouts [5, 6], matches the features of the grey prediction model well, which does not require much information and has simple calculations and high precision [7, 8]. Developed by Deng in 1982 [9], the grey theory deals with limited sample sizes (typically four or more samples) and poor information problems by employing ordinary differential equations to characterize the factors that may affect forecast accuracy. Since it provides a way to describe unknown systems under limited data conditions, it is widely used in engineering, economics, and science [10, 11]. Nonetheless, the grey model may yield less desirable performance when dealing with highly fluctuating data sequences during long-term prediction [12, 13].

In order to deal with datasets with more fluctuation like that of traffic crashes in roundabouts, we propose the integration of grey model techniques with Markov chains. Such a technique has already been proven to perform well with sparse and stochastic datasets with regards to determining causality and predicting future values. For example, Jin et al. [14] and Zou et al. [15] developed a one-factor Grey-Markov model to estimate the number of fatalities and economic losses associated with traffic accidents. These models, however, are all first-order grey models that takes into account only one independent variable. To date, few studies have given methods to determine the optimal dimension variables in multivariate Grey-Markov model prediction with limited data.

Driven by this question, this paper aimed to demonstrate the effectiveness of an optimized multivariable Grey-Markov model (MGM(1,n)-Markov) in fitting and predicting a stochastic and sparse roundabout crash dataset by comparing it with the classical GM(1,1) (grey model with one independent variable and one dependent variable) and optimized MGM(1,n) (grey model with multiple independent variable) models previously published in the literature [16]. The hybrid model consists of four steps. The first step involves performing a grey relational analysis using a dataset of traffic crashes at roundabouts from the Michigan Traffic Crash Facts dataset to measure the correlation between variables and the occurrence of traffic-related injuries. The next step is constructing a multivariate grey model for predictive theory by identifying the main elements or causal features describing collapse using grey relation analysis. As a third step, the residual error of the grey model prediction is modified using Markov theory, and as a final step, future trends are predicted.

The novelty aspects of this paper are summarized as follows:

1. Few studies have previously been published on the quantitative analysis of causality factors in roundabout traffic crashes.
2. We apply grey relational analysis to identify the weights and co-linearity between factors associated with accident occurrence in roundabouts, and combined with the Markov optimization model corrects the non-stationary data sequences.
3. A multivariable Grey-Markov model with sparse and stochastic data samples is proposed, which has not been applied to the prediction of roundabout traffic accidents.

To the best of our knowledge, there are only a few attempts to predict traffic accidents using multidimensional time series of influential factors. Most previous studies, particularly those focusing on roundabout traffic accidents, only examined a single variable, the number of accidents, and did not consider the traffic system’s multi-factor effects. Therefore, we aim to propose a multi-scale time-series roundabout crash prediction model that incorporates several human, vehicle, and road risk factors instead of relying solely on crash totals.

The remaining sections of the paper are organized as follows. We provide an overview of the state of the art of research in roundabout safety in the “Related works” section. The “Materials and methods” section presents the details of our methodology, which incorporates an optimized, multivariable MGM(1,n) model and Markov chains to update and optimize the grey model’s predictive results. The “Results” section presents the results of utilizing this methodology to model roundabout traffic crashes in Michigan. Consequently, these results, methodological considerations, mechanisms, practical application recommendations, and limitations are discussed in the “Discussion” section. Finally, the “Conclusions and perspectives” section provides a conclusion to this paper.

## Related works

In this section, we provide an extensive synthesis of the existing literature on the prevailing trends in roundabout development, emphasizing the application of predictive models for traffic crash forecasting.

Modern roundabouts, originating in the United Kingdom, have been designed to enhance traffic safety and flow [17]. While early designs presented constraints, introducing innovative traffic rules has improved capacity and safety. Research findings highlight the significant advantages of roundabouts for intermediate traffic demands, while signalized intersections exhibit superior performance in high-traffic scenarios [17, 18]. Roundabouts encourage low speeds, promoting safety, but possess unique crash profiles. Their implementation in the United States has led to decreased injury collisions and improved efficiency. However, the direct applicability of international research findings to the US context may be constrained due to various factors [18].

Current studies on traffic crash prediction focus on highways [6, 19–22], signalized intersections [23–25], and other roads [26, 27], whereas there has been little research on roundabouts in recent decades. Many approaches have been developed to solve traffic accident prediction problems, roughly categorized into three types: parametric models [28, 29] (e.g., ARIMA, grey prediction, spectral analysis), non-parametric models [6, 20, 21, 30–34] (e.g., non-parametric regression model, SVR, VAR, KNN, deep learning, parallel fp-growth mining, and types of neural network), and hybrid models [16, 22, 35] (e.g., Bayesian-neural network, fuzzy rule-based method, grey-ARIMA, and chaos-wavelet analysis-SVM). It is necessary to note that the methods above for predicting traffic collisions have higher requirements on the data sample size for training and verification and cannot capture data samples with considerable randomness [12]. However, the fact that roundabouts are an emerging mode of transportation in the United States and are not as widely deployed as in certain other countries determines that any available police-reported crash data would be highly sparse and stochastic. There is a gap in the current literature on a specific methodology to draw meaningful conclusions from such data samples.

Despite some progress in the above prediction models, certain research gaps in the existing literature still need to be explored in further detail. First, previous studies have overwhelmingly focused on conventional intersections, and relatively few studies have examined new roundabouts that may relieve traffic congestion to a great extent. Second, traditional prediction models commonly require normal data distribution and restrictions on sample size [8, 10]. In practice, roundabout traffic accidents consist of time series data that are highly nonlinear and scattered, making it difficult for historical data to be modeled and limiting prediction accuracy. Therefore, the key to accurate forecasting is choosing an appropriate method, especially in cases of incomplete data. Finally, relatively few studies on roundabout crashes have considered aggregated, empirical crash counts without adequately addressing the contributing explanatory variables [14, 36]. Numerous factors affect or explain such collisions, including driver behavior, weather conditions, traffic flow, and road structure [37–39]. Inevitably, most of these data-driven approaches are summary-statistics-based joint analyses, which are generally challenging to specify the insights of the prediction results. Such a literature gap calls for further research incorporating driver, vehicle, and environmental factors to analyze and predict roundabout traffic collisions with limited data.

## Materials and methods

### Data source

We used the data source of MTCF (Michigan Traffic Crash Facts) [40] yearly incidence data for roundabout traffic crashes from 2016 to 2021 to compile the roundabout traffic crash data in this paper. Note that this third-party data source is compiled by the Michigan State Police Office of Highway Safety Planning (OHSP) and the University of Michigan Transportation Research Institute (UMTRI) from all police reports of traffic crashes in the state of Michigan; and the authors of this article do not have and cannot provide direct access to the raw dataset. However, the dataset, as well as detailed breakdowns of crash figures by year and other explanatory variables, are available through MTCF’s public query tool at https://www.michigantrafficcrashfacts.org/querytool. Time frame-wise, we focus on traffic crashes from 2016 to 2021 since earlier records did not identify whether a collision occurred within a roundabout. Individual data items within this dataset were digitized from police reports of accidents.

According to Liu et al. [41], index screening should follow three principles, namely measurability, representativeness, and comparability. In accordance with this principle, we then selected eight explanatory variables (i.e., snow-covered, head-on crash on left turn, sideswipe, distraction, injury, median, buses/trucks, and rainy) to reflect the severity and probability of traffic accident risk (see Table 1). These eight variables are the only ones that provide continuous samples of non-trivial and non-zero data over the time period, given the sparsity and stochasticity of the data sample to begin with. Using this data set, we investigate the impact of each contributing element on the annual number of crashes at roundabouts compared to traditional intersections.

**Table 1:** Explanatory variables of traffic crash data in Michigan roundabouts.

Year	Total crashes	*X*_1_	*X*_2_	*X*_3_	*X*_4_	*X*_5_	*X*_6_	*X*_7_	*X*_8_
2016	489	20	1	146	23	48	5	25	52
2017	1510	73	3	535	48	125	19	97	154
2018	1501	78	3	523	41	116	16	86	112
2019	1864	85	5	642	57	146	19	162	176
2020	1300	61	2	416	49	103	19	96	77
2021	1730	79	1	618	56	132	23	131	116

Note that in the Table 1 above, *X*_1_ refers to crashes occurring on snow-covered roads, *X*_2_ refers to head-on crashes against another vehicle performing a left turn, *X*_3_ refers to a sideswipe crash, *X*_4_ refers to crashes with distracted drivers, *X*_5_ refers to crashes resulting in injuries, *X*_6_ refers to crashes in the median of roads, *X*_7_ refers to crashes involving buses and/or trucks, and *X*_8_ refers to crashes in rainy weather.

### Methodology overview

**Fig 1:**
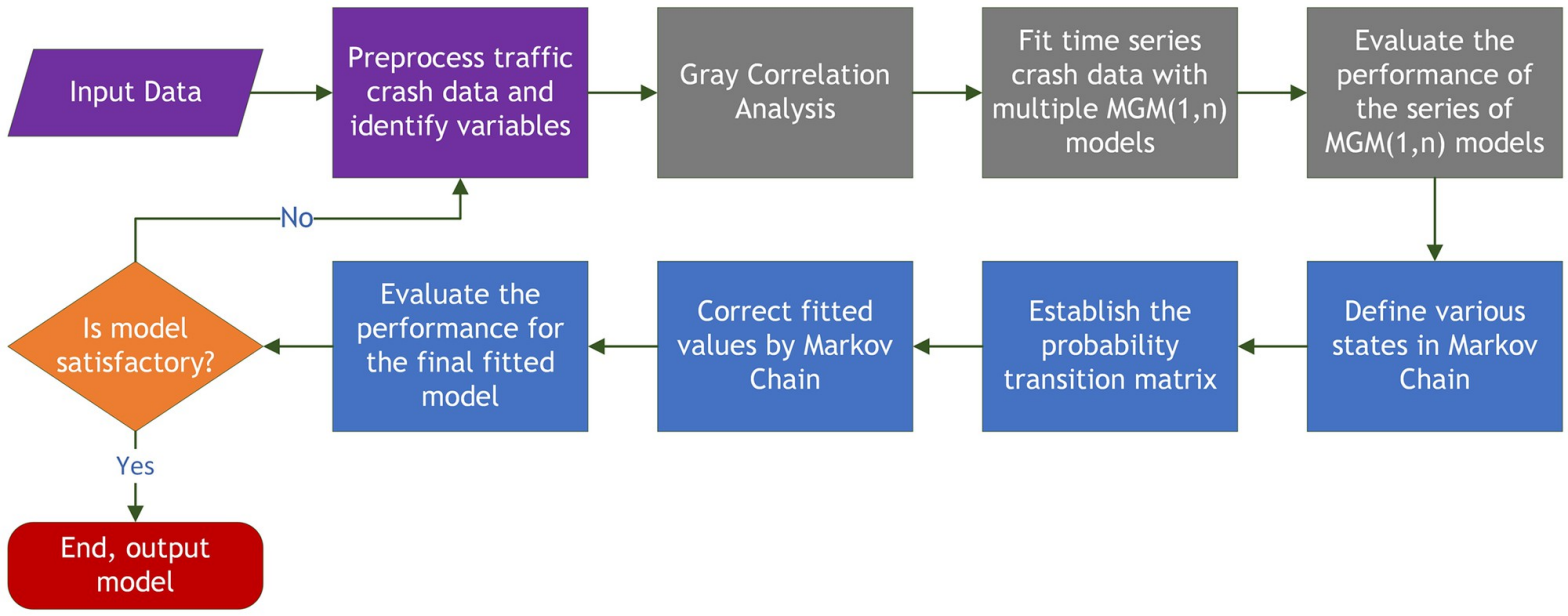
Flow chart of the optimized multivariate Grey-Markov model.

The purpose of this section is to propose an optimized multivariate Grey-Markov model for predicting short-term roundabout traffic crashes. Specifically, it combines grey relational analysis, the Multivariate Grey-Markov model, and the model evaluation framework for our predictor of traffic accidents (see Fig 1).

### Grey relational analysis

The basic idea of grey relational analysis is to judge the closeness of the relationship based on the similarity of the geometric shapes of the sequence curves. The higher the similarity, the greater the correlation. Traditional statistical methods have many drawbacks, such as requiring large amounts of data, typical sample distributions, and difficulty meeting practical needs. Grey relational analysis overcomes these shortcomings and only requires four data points without the need to meet typical distribution rules.

In many instances, roundabout accidents are caused by multiple potentially inter-connected factors. At the same time, numerous variables affect the occurrence and characteristics of accidents. We are interested in determining how each factor variable affects the accident variable to develop a reliable model for accident prediction. Typically, an impact factor for a potential accident is defined as a temporally ordered sequence of variables that characterize trends of the respective factor.

A variable sequence represents a time series of multiple variables that must be simulated and predicted to predict traffic accidents. We use grey relational analysis [42] to determine whether there is a compact connection between sequence curves based on their geometric similarity. Roundabout accident variables and their impact factors are com-pared to determine the coefficients.

Following grey relational analysis theory, let us suppose that a roundabout traffic system contains *m* time-series variables. The first of these variables describe the number of crashes, and the rest describe the various causality characteristics. Furthermore, a total of *n* measurements of those variables are taken at different discrete points in time. These time series can be represented as follows, where *i* refers to a certain time-series variable and *k* refers to one of the measurements of the variable over time.

$$\begin{array}{c} x_{i}=\left\{x_{i}\left(k\right)|k=1,2,\ldots ,n\right\}=\left\{x_{i}\left(1\right),x_{i}\left(2\right),\ldots ,x_{i}\left(n\right)\right\},i=1,2,\ldots ,m \end{array}$$ (1)

We can further define that *x*_1_ refers to the dependent time-series variable of traffic crash numbers, and that *x*_*j*_, *j* = 2, 3, …, *m* refers to the various independent causality characteristics that may be correlated to the outcome of traffic crash statistics [43]. Then the grey correlation between the number of crashes observed (*x*_1_) and an independent variable (*x*_*i*_) can be expressed as *r*_*i*_ in the following expression.

$$\begin{array}{c} r_{i}=\frac{1}{n}\sum_{k=1}^{n}\zeta_{i}\left(k\right),\;\text{where}\;\zeta_{i}\left(k\right)=\frac{{\text{min}}_{s}\;{\text{min}}_{t}|x_{1}\left(t\right)-x_{s}\left(t\right)|+\rho \;{\text{max}}_{s}\;{\text{max}}_{t}\left|x_{1}\left(t\right)-x_{s}\left(t\right)\right|}{x_{1}\left(k\right)-x_{i}\left(k\right)+\rho \;{\text{max}}_{s}\;{\text{max}}_{t}\left|x_{1}\left(t\right)-x_{s}\left(t\right)\right|} \end{array}$$ (2)

Note that *ρ* ∈ [0, 1] refers to a “grey correlation coefficient,” *ζ*_*i*_(*k*) provides the grey correlation coefficient at time point *k* between *x*_0_ and *x*_*i*_, min_*s*_ min_*t*_|*x*_1_(*t*) − *x*_*s*_(*t*)| and max_*s*_ max_*t*_|*x*_1_(*t*) − *x*_*s*_(*t*)| denotes the two-level minimum and maximum discrepancy, between *x*_1_(*t*) and any of *x*_2_(*t*), …, *x*_*m*_(*t*), respectively. We can then define *r*_*i*_ to be the grey correlation degree coefficient. The higher the value of *r*_*i*_, the stronger the correlation is between *x*_1_ and *x*_*i*_.

### Multi-variable Grey modeling

#### Variable and sequence definition

Let us denote the dependent variable sequence from the previous section as $x_{1}^{\left(0\right)}$ and the independent variable sequences from the previous section as $x_{j}^{\left(0\right)}$ with *j* = 2, 3, …, *m*. Then given *i* = 1, 2, …, *m*, we can apply the first-order accumulated generation operation (1-AGO) to $x_{i}^{\left(0\right)}=\left\{x_{i}^{\left(0\right)}\left(1\right),x_{i}^{\left(0\right)}\left(2\right),\ldots x_{i}^{\left(n\right)}\right\}$ to obtain an “accumulated” times series $x_{i}^{\left(1\right)}$ as follows.

$$\begin{array}{c} x_{i}^{\left(1\right)}\left(k\right)=\sum_{p=1}^{k}\;x_{i}^{\left(0\right)}\left(p\right),k=1,2,\ldots ,n \end{array}$$ (3)

Meanwhile, we can then define the adjoining main generated sequence of $x_{i}^{\left(1\right)}$ as $x_{i}^{\left(2\right)}$ [44].

$$\begin{array}{c} x_{i}^{\left(2\right)}\left(k\right)=\frac{1}{2}(x_{i}^{\left(1\right)}\left(k\right)+x_{i}^{\left(1\right)}\left(k-1\right)),k=2,3,\ldots ,n \end{array}$$ (4)

We can then define the vector of all time-series variables, their 1-AGO forms, and their adjoining main sequences at the discrete time point *k* ∈ [1, *n*] as follows.

$$\begin{array}{c} X^{\left(0\right)}\left(k\right)≔{\left[x_{1}^{\left(0\right)}\left(k\right),x_{2}^{\left(0\right)}\left(k\right),\ldots ,x_{m}^{\left(0\right)}\left(k\right)\right]}^{T} \end{array}$$ (5)

$$\begin{array}{c} X^{\left(1\right)}\left(k\right)≔{\left[x_{1}^{\left(1\right)}\left(k\right),x_{2}^{\left(1\right)}\left(k\right),\ldots ,x_{m}^{\left(1\right)}\left(k\right)\right]}^{T} \end{array}$$ (6)

$$\begin{array}{c} X^{\left(2\right)}\left(k\right)≔{\left[x_{1}^{\left(2\right)}\left(k\right),x_{2}^{\left(2\right)}\left(k\right),\ldots ,x_{m}^{\left(2\right)}\left(k\right)\right]}^{T} \end{array}$$ (7)

In order to fit a Multivariable Grey Model to, and to predict the value of the time series variables of *X*^(0)^ and *X*^(1)^, we can also introduce the following two vectors of continuous time functions that correspond to the model fitted values with regards to *X*^(0)^ and *X*^(1)^, respectively.

$$\begin{array}{c} {\tilde{X}}^{\left(0\right)}\left(t\right)≔{\left[{\tilde{x}}_{1}^{\left(0\right)}\left(t\right),{\tilde{x}}_{2}^{\left(0\right)}\left(t\right),\ldots ,{\tilde{x}}_{m}^{\left(0\right)}\left(t\right)\right]}^{T} \end{array}$$ (8)

$$\begin{array}{c} {\tilde{X}}^{\left(1\right)}\left(t\right)≔{\left[{\tilde{x}}_{1}^{\left(1\right)}\left(k\right),{\tilde{x}}_{2}^{\left(1\right)}\left(t\right),\ldots ,{\tilde{x}}_{m}^{\left(1\right)}\left(t\right)\right]}^{T} \end{array}$$ (9)

#### Grey model equation system definition

The Multivariable Grey model can be defined by the following system of first-order differential equations.

$$\begin{array}{c} \frac{\text{d}{\tilde{X}}^{\left(1\right)}}{\text{d}t}=A{\tilde{X}}^{\left(1\right)}+B \end{array}$$ (10)

where

$$\begin{array}{c} A=[\begin{array}{cccc} a_{11} & a_{12} & \cdots & a_{1m}\\ a_{21} & a_{22} & \cdots & a_{2m}\\ \vdots & \vdots & \ddots & \vdots \\ a_{m1} & a_{m2} & \cdots & a_{mm} \end{array}],B=[\begin{array}{c} b_{1}\\ b_{2}\\ \vdots \\ b_{m} \end{array}] \end{array}$$

Note that the initial condition is defined as ${\tilde{X}}^{\left(1\right)}\left(1\right)≔X^{\left(1\right)}\left(1\right)$.

We can acquire the values of *A* and *B* as follows. Let us first define *α*_*i*_ as the *i*-th row vector from the matrix [*A*|*B*], or

$$\begin{array}{c} \alpha_{i}={\left[a_{i1},a_{i2},\ldots ,a_{im},b_{i}\right]}^{T},i=1,2,\ldots ,m \end{array}$$ (11)

We can then compute the values for each *α*_*i*_ through the following expression.

$$\begin{array}{c} \alpha_{i}={\left(P^{T}P\right)}^{-1}P^{T}Q_{i} \end{array}$$ (12)

where

$$\begin{array}{c} P=[\begin{array}{cccc} x_{1}^{\left(2\right)}\left(2\right) & x_{2}^{\left(2\right)}\left(2\right) & \ldots & x_{m}^{\left(2\right)}\left(2\right)\\ x_{1}^{\left(2\right)}\left(3\right) & x_{2}^{\left(2\right)}\left(3\right) & \ldots & x_{m}^{\left(2\right)}\left(3\right)\\ \vdots & \vdots & \ddots & \vdots \\ x_{1}^{\left(2\right)}\left(n\right) & x_{2}^{\left(2\right)}\left(n\right) & \ldots & x_{m}^{\left(2\right)}\left(n\right) \end{array}],Q_{i}=[\begin{array}{c} x_{i}^{\left(0\right)}\left(2\right)\\ x_{i}^{\left(0\right)}\left(3\right)\\ \vdots \\ x_{i}^{\left(0\right)}\left(n\right) \end{array}] \end{array}$$

#### Grey model solution

The explicit, analytical solution to Eq 10 can thus be expressed as follows.

$$\begin{array}{c} {\tilde{X}}^{\left(1\right)}\left(t\right)=e^{At}X^{\left(1\right)}\left(1\right)+A^{-1}\left(e^{At}-I\right)B \end{array}$$ (13)

where *I* is the *m* × *m* identity matrix, and the matrix exponential *e*^*At*^ can be computed using Taylor series decomposition as follows.

$$\begin{array}{c} e^{At}=I+\sum_{j=1}^{\infty }\;\frac{A^{j}t^{j}}{k!} \end{array}$$ (14)

Finally we can recover the prediction values for the time series variables using their predicted 1-AGO forms. For instance, the following expression can be used if one wishes to predict the value of ${\tilde{x}}^{\left(0\right)}\left(m+1\right)$ given the predicted time series data for the first *m* time points.

$$\begin{array}{c} {\tilde{X}}^{\left(1\right)}\left(m+1\right)=e^{A(m+1)}X^{\left(1\right)}\left(1\right)+A^{-1}\left(e^{A(m+1)}-I\right)B \end{array}$$ (15)

$$\begin{array}{c} {\tilde{x}}^{\left(0\right)}\left(m+1\right)={\tilde{x}}^{\left(1\right)}\left(m+1\right)-{\tilde{x}}^{\left(1\right)}\left(m\right) \end{array}$$ (16)

### Markov chain modeling

In this section only, let *x* and $\tilde{x}$ denote the raw and predicted sequences of *n* variables obtained from the aforementioned Multivariable Grey Models, respectively.

#### State determination

We can first compute the following residual sequences between the raw and predicted data values.

$$\begin{array}{c} \delta =\left\{\delta \left(k\right)|k=2,3,\ldots ,n\right\},\;\text{where}\;\delta \left(k\right)=\frac{x\left(k\right)-\tilde{x}\left(k\right)}{\tilde{x}\left(k\right)} \end{array}$$ (17)

Then we can define the following Markov state transitions based on the above residual values. Note that each state *S*_*j*_ has a lower bound residual value of *L*_*j*_ and upper bound of *U*_*j*_, defined as follows.

$$L_{j}=\text{min}\left[\delta \left(i\right)\right]+\frac{j-1}{n}\left(\text{max}\left[\delta \left(i\right)\right]-\text{min}\left[\delta \left(i\right)\right]\right)$$ (18)

$$U_{j}=\text{min}\left[\delta \left(i\right)\right]+\frac{j}{n}\left(\text{max}\left[\delta \left(i\right)\right]-\text{min}\left[\delta \left(i\right)\right]\right)$$ (19)

#### State transition matrix

In order to correct for the inaccuracies of the output values $\tilde{\text{x}}$ of the Multivariable Grey Model, we would apply a linear transformation to each element of $\tilde{x}$ depending on which Markov state its residual value falls under. We can then define a *n*×*n* Markov transition probability matrix that defines such linear transformations.

$$\begin{array}{c} P\left(k\right)=[\begin{array}{cccc} P_{11}\left(k\right) & P_{12}\left(k\right) & \ldots & P_{1n}\left(k\right)\\ P_{21}\left(k\right) & P_{22}\left(k\right) & \ldots & P_{2n}\left(k\right)\\ \vdots & \vdots & \ddots & \vdots \\ P_{n1}\left(k\right) & P_{n2}\left(k\right) & \ldots & P_{nn}\left(k\right) \end{array}],\;\text{where}\;P_{ij}\left(k\right)=\frac{Q_{ij}\left(k\right)}{Q_{i}\left(k\right)} \end{array}$$ (20)

*P*_*ij*_(*k*) in the above matrix, named the “*k*-step transition matrix,” represents the probability for the residual of the predicted time series variable at time *m* + *k* to transition to *S*_*j*_ from the *S*_*i*_ state attained at time *m*. *Q*_*ij*_(*k*) represents the number of state transitions from *S*_*i*_ to *S*_*j*_ already observed from time *m* to *m* + *k* for any *m*. *Q*_*i*_(*k*) represents the number of observations where the residual falls within state *S*_*i*_.

It is also worthwhile noting that each of the row vectors of *P*(*k*) produces a sum up to 1, or that ∑_*j*_ *P*_*ij*_(*k*) = 1.

#### Correction of predicted value

Finally, for the Multivariable Grey Model output $\tilde{x}\left(k\right)$ whose residual is determined to be at state *S*_*j*_ = [*L*_*j*_, *U*_*j*_], we can produce an updated prediction value of $\tilde{y}\left(k\right)$ as follows [45].

$$\begin{array}{c} \tilde{y}\left(k\right)=\tilde{x}\left(k\right)(1+\frac{L_{j}+U_{j}}{2}) \end{array}$$ (21)

### Model evaluation

The proposed method is evaluated on a prediction task to compare the proposed modeling method with other alternative methods to compare its performance. In this process, we use different numeric metrics to measure the model fit and prediction accuracy, including mean absolute percentage error (MAPE), mean absolute error (MAE), and root mean square error (RMSE) [46].

The following are the formulae used to evaluate those specific metrics.

$$\begin{array}{c} MAPE_{i}\left(k\right)=\frac{1}{n}\sum_{k=1}^{n}|\frac{x_{i}\left(k\right)-{\tilde{x}}_{i}\left(k\right)}{x_{i}\left(k\right)}|\times 100\% \end{array}$$ (22)

$$\begin{array}{c} MAE_{i}\left(k\right)=\frac{1}{n}\sum_{k=1}^{n}\;|x_{i}\left(k\right)-{\tilde{x}}_{i}k|\times 100\% \end{array}$$ (23)

$$\begin{array}{c} RMSE_{i}\left(k\right)=\sqrt{\frac{1}{n}\sum_{k=1}^{n}{\left(x_{i}\left(k\right)-{\tilde{x}}_{i}k\right)}^{2}}\times 100\% \end{array}$$ (24)

Where *x*_*i*_(*k*) and ${\tilde{x}}_{i}\left(k\right)$ represented the actual and predicted time series variable values at time *k*, respectively.

## Results

This section presents the results of analyzing the Michigan Traffic Crash Facts dataset with the Grey-Markov modeling method as described in the previous section. The model fitness and its prediction performance are also to be compared against alternative models presented in the literature.

### Grey relational analysis

**Fig 2:**
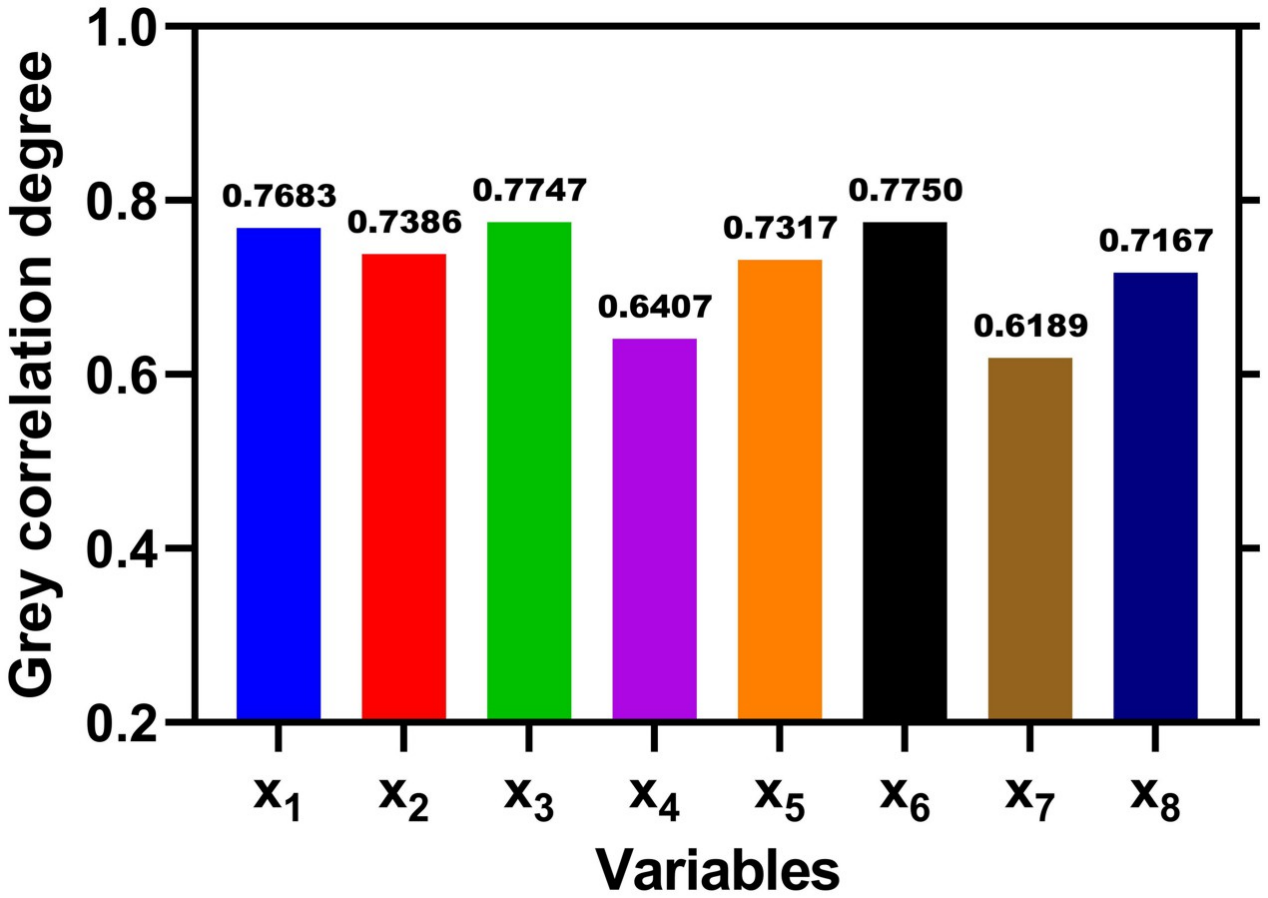
Grey relational grades of each independent variable associated with traffic crashes in roundabouts.

Before choosing appropriate forecast factors, we shall first calculate the grey incidence degree. The factors with distinct lower grey relational degrees will be omitted, reducing calculation and complexity to minimize the errors. According to Eq 2, and given synthetic coefficient *ρ* = 0.5 [42], the degrees of influence of each factor impacting the causality of crashes are shown as specified in the following Fig 2.

Similarly to Table 1, *X*_1_ refers to crashes occurring on snow-covered roads, *X*_2_ refers to head-on crashes against another vehicle performing a left turn, *X*_3_ refers to a sideswipe crash, *X*_4_ refers to crashes with distracted drivers, *X*_5_ refers to crashes resulting in injuries, *X*_6_ refers to crashes in the median of roads, *X*_7_ refers to crashes involving buses and/or trucks, and *X*_8_ refers to crashes in rainy weather.

The figure above shows us that the grey relation scores for roundabout accidents, driver distraction, and crashes involving buses/trucks are not high enough. In other words, factors with grey incidence values smaller than a set threshold of 0.7 can be omitted [42].

### Results of model selection

The potential numbers of causality factors to traffic crashes in roundabouts are admittedly numerous, and it would be unrealistic and inefficient to consider every possible factor in a single model. Therefore, we decide that only a certain number of factors that are enough correlated with the occurrence of traffic crashes in roundabouts are to be considered. Our metric for this degree of correlation is “grey correlation factor.” We also determined that only those causality factors with grey correlation factors over a threshold of 0.7 would be considered as the “main factors.”

**Fig 3:**
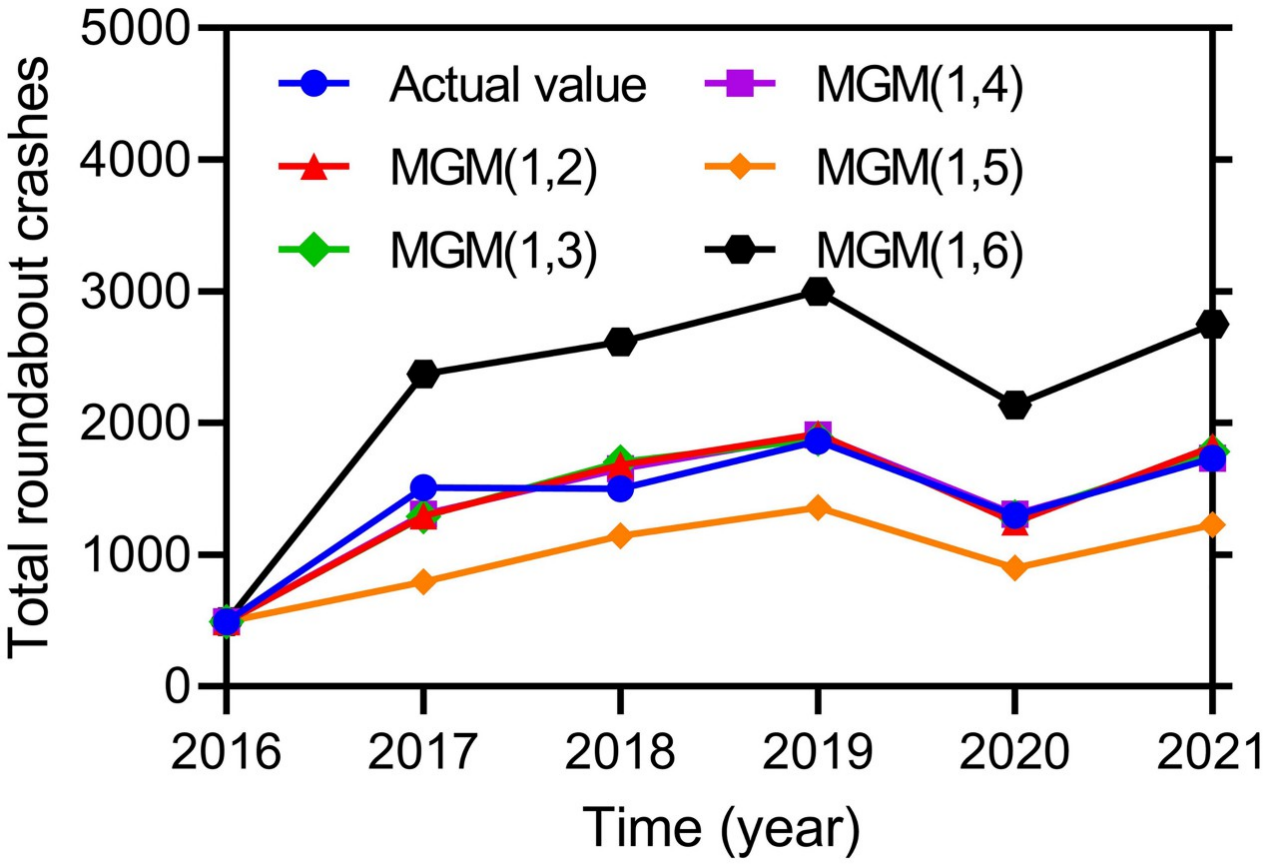
Fitted accuracies of different Multivariable Grey (MGM(1,n)) models.

Different Multivariable Grey (MGM(1, *n*)) models can be constructed with any number (*n*) of independent variables that have the highest ranking in their correlation grades, as provided in the previous section. In order to obtain the ideal number of independent variables for fitting our dataset, we can construct multiple Multivariable Grey models using a range of independent variables from 2 to 6. The MAPE percentage errors are computed for each model for each of the fitted values that it outputs as shown in Eq 22. The results of this evaluation process is shown below in Fig 3.

Similarly to Table 1, *X*_1_ refers to crashes occurring on snow-covered roads, *X*_2_ refers to head-on crashes against another vehicle performing a left turn, *X*_3_ refers to a sideswipe crash, *X*_4_ refers to crashes with distracted drivers, *X*_5_ refers to crashes resulting in injuries, *X*_6_ refers to crashes in the median of roads, *X*_7_ refers to crashes involving buses and/or trucks, and *X*_8_ refers to crashes in rainy weather.

Also note the following:

1. The input parameters for the MGM(1,2) model include *X*_3_, *X*_6_.
2. The input parameters for the MGM(1,3) model include *X*_1_, *X*_3_, *X*_6_.
3. The input parameters for the MGM(1,4) model include *X*_1_, *X*_2_, *X*_3_, *X*_6_.
4. The input parameters for the MGM(1,5) model include *X*_1_, *X*_2_, *X*_3_, *X*_5_, *X*_6_.
5. The input parameters for the MGM(1,6) model include *X*_1_, *X*_2_, *X*_3_, *X*_5_, *X*_6_, *X*_8_.

Due to the collinearity between different variables and the redundancy among variables, the MGM(1,n) model will have difficulty making accurate predictions due to confounding variables exerting other effects depending on the interference. Similarly, the most influential factors largely reflect the roundabout traffic collapse system changes. Referring to Fig 3, we then calculated the MAPE for MGM(1,2), MGM(1,3), MGM(1,4), MGM(1,5), and MGM(1,6) model as 6.39%, 5.50%, 4.47%, 26.43%, and 52.56%, respectively. Thus, the MGM(1,4) model with the highest prediction accuracy was selected as the best representation of the influential variables based on a comparison of prediction accuracy across models.

### Hybrid, optimized MGM(1,4)-Markov model

A hybrid model was further developed based on the MGM (1,4) model. According to the relative error (*R*_*E*_) between the actual value and the fitted value, we divided the relative error into three state intervals:

The state *S*_1_ signifies underestimation of the fitted value, with the relative error satisfying the following condition.

$$\begin{array}{c} \text{min}\left(R_{E}\right)\leq R_{E}\leq \text{min}\left(R_{E}\right)+\frac{\left|\text{min}(R_{E})|+|\text{max}(R_{E})\right|}{3} \end{array}$$ (25)

The state *S*_2_ is the normal state of the fitted value, with the relative error satisfying the following condition.

$$\begin{array}{c} \text{min}\left(R_{E}\right)+\frac{\left|\text{min}(R_{E})|+|\text{max}(R_{E})\right|}{3}\leq R_{E}\leq \text{max}\;R_{E}-\frac{\left|\text{min}(R_{E})|+|\text{max}(R_{E})\right|}{3} \end{array}$$ (26)

The state *S*_3_ signifies overestimation of the fitted value, with the relative error satisfying the following condition.

$$\begin{array}{c} \text{max}\;R_{E}-\frac{\left|\text{min}(R_{E})|+|\text{max}(R_{E})\right|}{3}\leq R_{E}\leq \text{max}\;R_{E} \end{array}$$ (27)

According to the relative error of the actual value to the fitted value, three statuses were identified: *S*_1_ ≔ [−0.1013, −0.0229), *S*_2_ ≔ [−0.0229, 0.0554), and *S*_3_ ≔ [0.0554, 0.1338]. The status divisions from the 2016 to 2021 series are shown in Table 2.

**Table 2:** Markov states of MGM(1,4) output values.

Year	Actual value	MGM(1,4)	Relative error	State
2016	489	489	0.0000	*S*_2_
2017	1510	1308	0.1338	*S*_3_
2018	1501	1653	-0.1013	*S*_1_
2019	1864	1910	-0.0247	*S*_1_
2020	1300	1308	-0.0062	*S*_2_
2021	1730	1734	-0.0023	*S*_2_

Based on the above state distributions, we can compute the following 1-, 2-, and 3-step probability matrices of Markov state transitions, as specified in Eq 22.

$$\begin{array}{c} P\left(1\right)=[\begin{array}{ccc} \frac{1}{2} & \frac{1}{2} & 0\\ 0 & \frac{1}{2} & \frac{1}{2}\\ 1 & 0 & 0 \end{array}],P\left(2\right)=[\begin{array}{ccc} \frac{1}{4} & \frac{1}{2} & \frac{1}{4}\\ \frac{1}{2} & \frac{1}{4} & \frac{1}{4}\\ \frac{1}{2} & \frac{1}{2} & 0 \end{array}],P\left(3\right)=[\begin{array}{ccc} \frac{3}{8} & \frac{3}{8} & \frac{1}{4}\\ \frac{1}{2} & \frac{3}{8} & \frac{1}{4}\\ \frac{1}{4} & \frac{1}{2} & \frac{1}{4} \end{array}] \end{array}$$ (28)

Based the a 3-step transition probability matrices from Eq 28, we can compute the fol-owing predicted state transitions of the yearly roundabout crash numbers as in Table 3. Our calculations are based on the three most recent values and use different transfer steps to calculate the predicted values.

**Table 3:** Predicted Markov state transitions of yearly traffic crash figures.

Year	Initial state	Transfer step	*S*_1_	*S*_2_	*S*_3_
2021	*S*_2_	1	0	1/2	1/2
2020	*S*_2_	2	1/2	1/4	1/4
2019	*S*_1_	3	3/8	3/8	1/4
Total			7/8	9/8	1

According to Table 3, the traffic crashes at Michigan roundabouts in 2022 were most likely in *S*_2_. Accordingly, the predicted revised GM(1,1)–Markov chain value in 2022 based on the above prediction steps can be obtained as follows.

$$\begin{array}{c} \tilde{y}=\left(1+0.5\times \left(-0.029+0.0554\right)\right)\times 1730\approx 1758 \end{array}$$ (29)

Our study suggests that the number will likely increase slightly next year, implying that taking steps to minimize the likelihood is important.

### Model comparison

**Fig 4:**
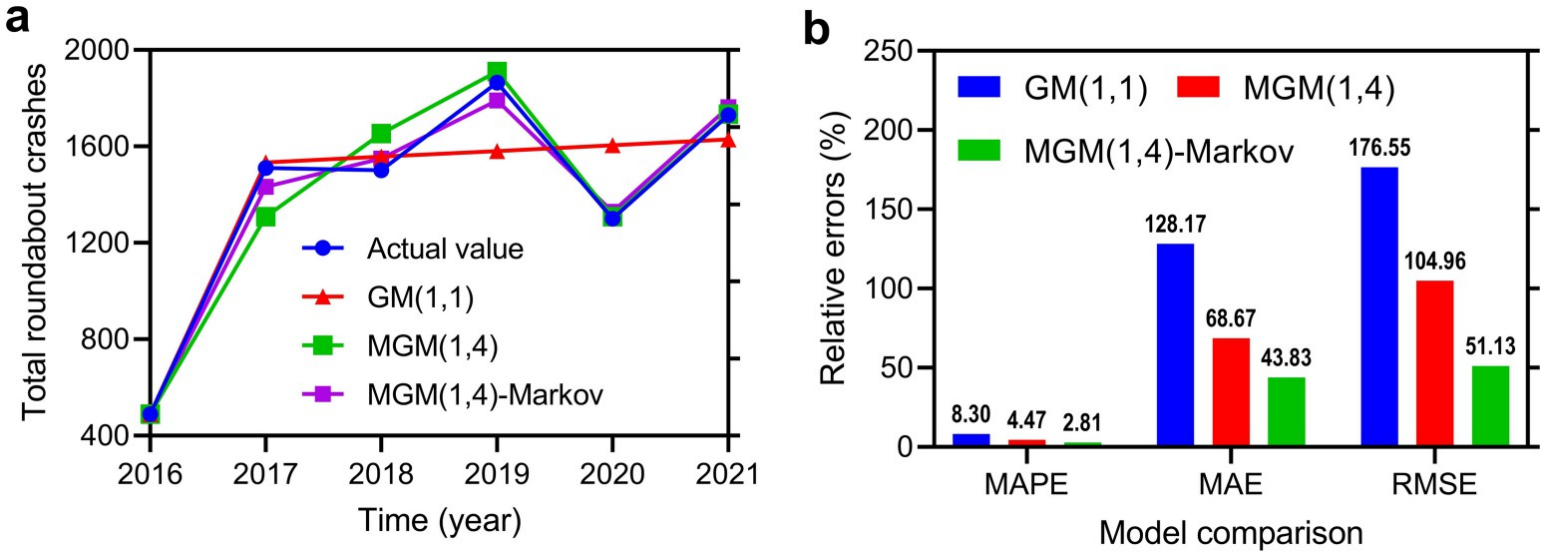
Comparison between existing GM [47] and MGMs [48] in literature and the MGM-Markov model. (a) Model-fitted total roundabout crashes; (b) Relative error values.

A hybrid approach combining the theory of grey systems and Markov chains has been used to predict roundabout accidents. Time series data from 2016 to 2021 were fitted using the GM(1,1) model [47], MGM(1,4) model [48], and hybrid MGM(1,4)-Markov model, as shown in Fig 4.

According to the results, the average MAPE values of GM(1,1) models, MGM(1,4) models, and Grey-Markov models were 8.30%, 4.47%, and 2.81%, respectively. The MAE values of the GM(1,1), MGM(1,4), and Grey-Markov models were 128.17%, 68.67%, and 43.38%, respectively. The RMSE values of the GM(1,1), MGM(1,4), and Grey-Markov models were 176.55%, 104.96%, and 51.13%, respectively. The fitting results indicate that the grey Markov model outperforms the single grey prediction in accuracy. When applying the grey model to compare actual incident data with the predicted values, we find that the relative errors are high and incongruous. However, after correcting the predictions of the grey model using a Markov chain process, we can enhance the prediction accuracy by combining multiple prediction models. Thus, the hybrid Grey-Markov model is more suitable for predicting the number of incidents from 2016 to 2021.

## Discussion

To our knowledge, this is one of the first attempts to predict roundabout traffic crashes via an optimized multivariate Grey-Markov model. Our proposed MGM(1,n)-Markov model represents a valid and efficient method for predicting round-about traffic crashes in Michigan under insufficient data, temporal fluctuations, and trend changes. In summary, the findings of this study may assist policymakers in formulating policies to improve roundabout traffic safety and potentially reduce crashes. Given the results of this study, the remainder of this section elaborates on gaps in the existing literature and priority areas that should be addressed in future studies of traffic crashes at roundabouts.

### Summary of evidence

First, we identify eight roundabout traffic crash impact factors and then apply the grey relational algorithm to prioritize these impact factors. Roundabout traffic accident risk can be assessed from various perspectives using these indicators, which have proven effective. Roundabout traffic crashes are influenced by multiple sources of information, including humans, vehicles, and surrounding elements, each of which interacts with the others and influences the outcome [3, 4, 22, 39]. Meanwhile, annual traffic crash prediction can be viewed as a problem associated with the grey system because several factors (i.e., humans, vehicles, and surroundings) influence roundabout traffic crashes, but the precise relationships between these factors are, at least within the scope of this study, unclear. Among the factors influencing roundabout traffic accidents in Michigan, crashes on the median strip (grey correlation 0.8186) have the greatest impact. The constructed model can predict these factors with high accuracy. The traffic administration can use the results of this study to control the impact factors and reduce the likelihood of traffic accidents at roundabouts.

Second, we found that multivariate optimization (MGM(1,n)) outperforms uni-variate optimization (GM(1,1)) because multiple variables are simultaneously considered. Our analysis indicates that the one-dimensional GM(1,1) model performed well with a good prediction, while the optimized four-dimensional MGM(1,4) model performed much better with a lower average relative error. Hybrid MGM(1,4)-Markov models can better fit the nonlinear functions of raw time sequences than single grey models and MGM(1,4) models, making them effective tools for preventing traffic accidents at roundabouts. There is evidence that the hybrid MGM (1,4)-Markov model produced more accurate predictions and that the forecasted results may prove useful in assisting management in dealing with traffic issues. A Markov chain is well-suited to deal with intra-sequence fluctuations since the grey model is well-suited to exponential sequences. The results of this study indicate that the hybrid model is more accurate in terms of fitting data than the basic GM(1,1) model. Despite the reduction in accuracy, the Markov model provides interval-based predictions and improves prediction accuracy. In general, four-dimensional MGM(1,n) is a more accurate prediction model based on relevant variables’ influence than other models.

Finally, Markov chains have been proven to be a valid methodological approach to enhance the fitting accuracy of the MGM(1,n) model through modification of the prediction results. A Markov chain process is characterized by the fact that it does not require a large amount of data; Only a limited amount of information is needed to make a prediction [49]. This may be because a Markov probability matrix can be used to determine the transfer rules of states, while the MGM(1,n) model can reveal the predicted data’s development trend. With the combination of the two models, the resulting information from the raw data can be adequately evaluated, resulting in high prediction accuracy and stochastic volatility. Prior studies [43, 50] has focused primarily on optimizing background values; however, residual modification models are rarely applied to the MGM(1,n) model. The prediction accuracy of traditional residual modification models has been improved by developing modified residual modification models such as the Grey model, Markov chains, Fourier series, genetic programming, and neural networks. Based on comparisons of the results of the MGM(1,n)-Markov model, the GM (1,1) model, and the optimized MGM(1,n) model, the first model is more accurate, with a prediction error of 3.02%, a much smaller error than the alternative models of 8.30% and 4.47%, which provides evidence that the method is feasible. To date, only a few existing studies, such as Zouhair et al. [51] and Govindan et al. [52], the Grey-Markov predictive models, have been used to predict maritime incidents and traffic volumes.

### Interpretation of results

Predicting roundabout accidents based on limited data is essential in traffic management and decision-making when prioritizing road safety projects. There have been some qualitative or quantitative studies of the factors contributing to these accidents in the past, but a clear and complete picture is difficult to obtain due to incomplete information and additional factors that interact [53]. The following reasons may explain these findings.

The first interpretation concerns exploratory studies with limited sample sizes. This is mainly due to the dynamic and time-varying nature of roundabout traffic systems and finite volume data, indicating a non-stationary stochastic process with definite trends. Typically, previous models require a certain amount of historical data to make predictions, which are not guaranteed to be accurate with limited data [54]. Therefore, it is not possible to use these models to predict road accidents, as they are not suitable for that purpose. Moreover, models based on older data are less accurate and add uncertainty than models based on more recent data, resulting in reduced estimation accuracy [55]. In future studies, it would be beneficial to consider ideal data collection times for the grey model.

The second explanation involves factors that affect predictive accuracy, which was complex before. A grey relational analysis of traffic crashes shows that the importance of the grey relational rank differs between reference and comparison series. This suggests that additional influences influence roundabout traffic crashes, and each influence plays a distinct role in influencing these crashes. Interactions between humans, vehicles, and surroundings also lead to more uncertainty in the prediction results and large data fluctuations. On the other hand, we found that a single grey model (i.e., GM(1,1) and MGM(1,n)) is not optimal in terms of prediction accuracy. In this case, this can be explained by the model requiring exponential regularity for the data sequence, and a relatively high degree of stochastic fluctuations is not acceptable [12, 13].

The third interpretation involves a difference in methodology considerations. It is possible to characterize roundabout traffic accidents as time series data affected by human interaction, vehicles, and surrounding factors. Choosing a suitable forecasting algorithm is vital to traffic accident forecasting. The traditional forecasting methods (e.g., parametric models [28, 29] and non-parametric models [20, 30–33, 56]) play an essential role in actual work; however, there are limitations due to the distribution and size of the sample. While grey prediction can overcome insufficient data-driven shortcomings of traditional estimation models, it is relatively poor at predicting roundabout traffic crashes, which are largely fluctuating data series influenced by humans, vehicles, and surroundings. It has been demonstrated that Markov chains apply to large fluctuations in data series; thus, by combining them with the grey prediction model, limitations due to a lack of data and changes in the raw data sequence can be overcome. The usage of its forecast for roundabout traffic crashes can deal with data sequences that fluctuate highly, as well as takes into account the interaction factors (e.g., humans, vehicles, surroundings) with a great scientific and practical method. Accordingly, the hybrid MGM(1,n)-Markov model has a high level of accuracy in its prediction for roundabout traffic crashes from 2016 to 2021 (Fig 3).

Our finding was that the single GM(1,1) or MGM(1,n) models were found to have a large relative error of prediction in some years, reflecting the fluctuation of roundabout traffic crashes. However, after error testing, the model’s accuracy was qualified, indicating that it is suitable for predicting roundabout traffic accidents. The error in the prediction value needs to be corrected. A hybrid Grey-Markov chain model is constructed, and the Markov chain method is used to modify the predictions after making them. As a result of reducing prediction error and performing an accuracy test, we found that the Grey-Markov chain model can make more accurate predictions. Our results suggest that the Grey-Markov chain model performs significantly better than the single GM(1,1) model, as shown in Fig 3. Studies have shown that the Markov chain achieves high accuracy by correcting predictions’ fitted curves to match the fluctuations of the actual values.

Nevertheless, a note should be made regarding the number of state partitions included in the Grey-Markov model. State divisions determine the accuracy of their predictions, which are not standardized but depend on the amount of historical data. More states should be split if there is a large amount of historical data, resulting in greater prediction accuracy. Large state divisions have enhanced prediction accuracy, yet, too many states also lead to small samples per state and low transition probabilities. Therefore, a reasonable division of states number is recommended to improve prediction accuracy.

## Conclusions and perspectives

This paper constructed a hybrid-optimized Grey-Markov model and compared it with two additional existing models to predict roundabout traffic crashes in Michigan from 2016 to 2021. The optimized multivariate Grey-Markov model outperforms other grey models for predicting roundabout traffic accidents, achieving higher prediction accuracy. We can also conclude that similar approaches to that undertaken in this study can become suitable for solving problems related to long-term prognosis and fluctuations in data, as demonstrated in the experiments.

A summary of the key findings of this study are listed as follows.

1. Roundabout traffic accidents can be caused by various factors, including humans, vehi-cles, and the surrounding environment. Our findings demonstrate that the leading major causes of accidents in Michigan roundabouts are ranked as follows: crash on median (0.7750), sideswipe (0.7747), snow-covered road (0.7683), head-on crash on left turn (0.7386), crash with injury (0.7317), crash in rainy weather (0.7167), distraction driver (0.6407), and buses/trucks crash (0.6189) through grey relational analysis.
2. In the context of roundabout traffic accidents, the proposed hybrid Grey-Markov model is suitable for short-term predictions with limited sample data. Grey system theory can handle uncertainty in the data, and Markov chain processes enhance its overall accuracy by correcting its predictions. For a limited sample of cases, the hybrid multivariate Grey-Markov model performs exceptionally well, demonstrating a close match between predicted and actual values. Thus, roundabout accident prediction with limited data is effectively applied with a mean relative error of 3.02%.
3. Several countermeasures have been developed in response to these risk factors affecting roundabout crashes, urging the government, society, and drivers to work together. Further studies in the vehicle region may benefit from examining more detailed incident data to identify causal relationships between roundabout collisions.

The proposed prediction method makes several practical contributions to roundabout traffic. First, we offered a hybrid roundabout traffic crash prediction model that combines grey systems theory and Markov chains, which has significant engineering applications to assist policymakers in predicting roundabout traffic accidents with limited data. To our knowledge, there have been no extensive studies of such combined prediction methods; Second, our proposed approach provides a detailed analysis of how impact factors relate to roundabout traffic crashes. The grey relational analysis shows that the top three causes of roundabout accidents in Michigan over the past six years were crashes in the median, head-on collisions in left turns, and sideswipes from the same direction. Therefore, authorities should consider the findings of this study when planning future traffic enforcement priorities for roundabouts, considering the limited number of enforcement officers available. Finally, the results provide a basis for preventing roundabout traffic crashes in the future and similar methodological applications (e.g., maritime, mining, and civil aviation accidents). In other words, this approach simultaneously examines human, vehicle, and surrounding factors and provides insights into fundamental issues regarding future roundabout traffic and practical countermeasures.

We propose the following countermeasures based on the characteristics and predictions of traffic collisions at roundabouts:

1. Governments should establish appropriate mechanisms to formulate specific policies regarding roundabout traffic safety based on the attributes of roundabouts and analyze the available data to extract valuable information.
2. Law enforcement should intensify their efforts at intersections by enforcing strict sanctions against violators to control the reckless behavior of drivers. Decision makers may benefit from the results of this study in dealing with roundabouts and other traffic control tools. This study should, however, be extended to further locations in Michigan with different characteristics.
3. Vehicle drivers should pay adequate attention to traffic safety. We found that driver inattention or distraction is one of the leading causes of roundabout accidents. Therefore, regular publicity must inform drivers about unsafe practices that can lead to roundabout accidents and serious consequences. Traffic safety must be taken very seriously, and all regulations must be strictly followed.

There are a few limitations that must be considered in this study. First, the roundabout crash data collected for this study were derived from official reports with limited observations, which could be biased, and the data should be interpreted cautiously. Due to the limited sample size, we may only be able to provide preliminary results since the information gathered may not be sufficient and a larger database on roundabouts is necessary. Second, roundabout traffic crashes are influenced by multiple factors, yet we only consider eight key variables. Further research will be conducted to incorporate additional roundabout traffic crash factors into the model and to improve the predictive model from the point of view of the mechanisms involved. Third, the hybrid Grey-Markov model is a predictive approach with limited data conditions, and further sensitivity analysis is necessary to determine the optimal data sample size and state partition to improve the prediction accuracy. Finally, the data are from Michigan and may not apply directly to settings in other states of the U.S. or other countries. Additionally, this improved model may be extended to alternative fields (e.g., maritime, mining, and civil aviation accidents); however, additional theoretical and applied studies are needed.

Despite the hybrid MGM(1,4)-Markov model performing well in predicting round-about traffic accidents, further expansion and verification of the grey forecasting model is expected in future studies. Further research is needed to combine other artificial intelligence approaches to solve uncertain system modeling for long-term forecasting effects. By expanding model construction and relaxing parameter setting and model construction restrictions, artificial intelligence can be used to compensate for the traditional grey model’s obvious linear feature, thus compensating for its shortcomings.
